# Recurrent activations of transient receptor potential vanilloid‐1 and vanilloid‐4 promote cellular proliferation and migration in esophageal squamous cell carcinoma cells

**DOI:** 10.1002/2211-5463.12570

**Published:** 2019-01-18

**Authors:** Rongqi Huang, Fei Wang, Yuchen Yang, Wenbo Ma, Zuoxian Lin, Na Cheng, Yan Long, Sihao Deng, Zhiyuan Li

**Affiliations:** ^1^ Key Laboratory of Regenerative Biology Guangdong Provincial Key Laboratory of Stem Cell and Regenerative Medicine Guangzhou Institutes of Biomedicine and Health Chinese Academy of Sciences Guangzhou China; ^2^ University of Chinese Academy of Sciences Beijing China; ^3^ Department of Anatomy and Neurobiology Xiangya School of Medicine Central South University Changsha China; ^4^ GZMU‐GIBH Joint School of Life Sciences Guangzhou Medical University China

**Keywords:** Ca^2+^ imaging, cellular migration, cellular proliferation, esophageal squamous cell carcinoma, TRPV

## Abstract

Some members of the transient receptor potential vanilloid (TRPV) subfamily of cation channels are thermosensitive. Earlier studies have revealed the distribution and functions of these thermo‐TRPVs (TRPV1–4) in various organs, but their expression and function in the human esophagus are not fully understood. Here, we probed for the expression of the thermo‐TRPVs in one nontumor human esophageal squamous cell line and two esophageal squamous cell carcinoma (ESCC) cell lines. TRPV1, TRPV2, and TRPV4 proteins were found to be upregulated in ESCC cells, while TRPV3 was not detectable in any of these cell lines. Subsequently, channel function was evaluated via monitoring of Ca^2+^ transients by Ca^2+^ imaging and nonselective cation channel currents were recorded by whole‐cell patch clamp. We found that TRPV4 was activated by heat at 28 °C–35 °C, whereas TRPV1 and TRPV2 were activated by higher, noxious temperatures (44 °C and 53 °C, respectively). Furthermore, TRPV1 was activated by capsaicin (EC_50_ = 20.32 μm), and this effect was antagonized by AMG9810; TRPV2 was activated by a newly developed cannabinoid compound, O1821, and inhibited by tranilast. In addition, TRPV4 was activated by hypotonic solutions (220 m Osm), and this effect was abolished by ruthenium red. The effects of TRPV1 and TRPV4 on ESCC were also explored. Our data, for the first time, showed that the overactivation of TRPV1 and TRPV4 promoted the proliferation and/or migration of ESCC cells. In summary, TRPV1, TRPV2, and TRPV4 were functionally expressed in human esophageal squamous cells, and thermo‐TRPVs might play an important role in the development of ESCC.

**Abbreviations**
CCK8: cell counting kit‐8
EC_50_: half maximal effective concentration
ESCC: esophageal squamous cell carcinoma
HBSS: Hank's balanced salt solution
HEPES: 4‐(2‐hydroxyethyl)‐1‐piperazineethanesulfonic acid
IC_50_: half maximal inhibitory concentration
Osm: osmotic pressure
RT‐PCR: reverse‐transcription polymerase chain reaction
TRPV: transient receptor potential vanilloid subfamily

In mammals, the transient receptor potential vanilloid (TRPV) subfamily consists of the six members TRPV1–TRPV6, among which the TRPV1–4 genes are related to warm sensing or thermal pain. These four TRPV channels are thermosensitive and can be activated by different temperature ranges; therefore, they are known as ‘thermo‐TRPVs’ 1, 2, 3. Thermo‐TRPV channels belong to the nonspecific cation channel receptor family; activation by heat or appropriate agonists will result in inward currents of multiple cations, including Na^+^ and especially Ca^2+^ 4, 5.

Transient receptor potential vanilloid 1, the first identified thermo‐TRPV channel, is a polymodal channel which can be activated by heat (>43 °C), capsaicin, protons (pH ≤ 5.9), cannabinoids, and endogenous lipids, resulting in calcium entry 6. TRPV1 is highly expressed in peripheral nerve terminals as well as in multiple non‐neuronal cell types 7, such as epidermal keratinocytes, liver cells, bladder urothelium, cells of the gastrointestinal tract, polymorphonuclear granulocytes, and macrophages 8. TRPV1 is now believed to function as a molecular integrator of noxious stimuli, including acids, heat, and endogenous pro‐inflammatory substances 7, 9. In dorsal root ganglion neurons, the TRPV1 channel plays an essential role in pain signal generation and regulation 10. High expression and/or overactivation of TRPV1 has been found to be involved in disease states of the digestive tract such as inflammatory bowel disease, irritable bowel syndrome, and esophagitis 2.

In contrast to TRPV1, the transient receptor potential vanilloid receptor 2 (TRPV2) is insensitive to capsaicin, acid, and moderate heat but does respond to higher temperature stimuli (≥52 °C) 11, which is the highest activation temperature threshold among all the thermo‐TRPVs. TRPV2 has also been shown to be sensitive to certain chemicals [such as 2‐aminoethoxydiphenyl borate (2‐APB), probenecid, and lysophospholipids], hypotonic solutions, and mechanical stimuli 12. Furthermore, TRPV2 is also activated by endogenous modulators such as insulin, insulin‐like growth factors, epidermal growth factor, and platelet‐derived growth factor 3. It appears that these ligands regulate TRPV2 mainly by inducing translocation of TRPV2 to the cellular membrane and increasing Ca^2+^ entry 13. TRPV2 is abundantly expressed in a subpopulation of sensory neurons that predominantly give rise to Aα fibers. In the central nervous system, TRPV2 is expressed in striatal, hippocampal, and hypothalamic neurons and may play an important role in the regulation of body fluid homeostasis, autonomic function, and metabolism 14. TRPV2 is also expressed within non‐neuronal cells and tissues, such as mast cells 11, 15, aortic smooth muscle cells, lung, spleen, and intestine tissues 2, 12, and cardiomyocytes 3. TRPV2 has been found to participate in the pathology of various types of human cancers, including breast tumors 16, prostate cancer 17, and multiple myeloma 4. It has been proposed as a prognostic marker in hepatocellular carcinoma 18.

Transient receptor potential vanilloid 3 and TRPV4 are activated by moderate temperatures, with thresholds of 34 °C–38 °C and 27 °C–34 °C, respectively 19. Mice lacking TRPV3 or TRPV4 have been reported to exhibit deficits in both innocuous and noxious heat sensation, indicating the involvement of both channels in thermosensation 9, 20. TRPV3 is a structural homologue of TRPV1, sharing 40–50% homology, and is co‐expressed in dorsal root ganglion neurons, as well as the skin, tongue, spinal cord, and brain with TRPV1 9, 21. It is sensitive to heat but insensitive to capsaicin 12. It can also be activated by chemicals such as diphenyl‐containing compounds, camphor, menthol, and 1,8‐cineol 22. TRPV3 is an important cutaneous sensor that detects thermal and chemical stimuli and, hence, is implicated in skin sensitization and hyperalgesia in inflamed tissues 21, 23. Increased expression of TRPV3 has been found in the case of peripheral nerve injury 19. TRPV3 is also present in corneal epithelial cells and plays a role in thermosensation and in the regulation of cell proliferation 24.

Transient receptor potential vanilloid 4 was first described as an osmosensor that detects hypotonic stimuli and shares 40% amino acid identity with TRPV1 25. TRPV4 can be activated by osmotic cell swelling, moderate heat (>27 °C), mechanical stimuli, the phorbol ester derivative 4α‐PDD (4α‐phorbol 12,13 didecanoate), and lipid metabolites 22, 26. Its low heat activation threshold implies an increased basal activity of TRPV4 at normal body temperature 19. TRPV4 is highly expressed in skin keratinocytes and epithelia lining tubular structures throughout the body. As such, it functions as a polymodal cellular sensor and is involved in many different cellular functions 9. TRPV4 has been reported to function as an osmotic sensor in the central nervous system and as a key molecule regulating neuronal excitability 25, also to be involved in temperature sensation and the integration of thermal and osmotic information 26. It has also been found to participate in the pathogenesis of acute lung injury and adult respiratory distress syndrome (ARDS) and to be an important target in the treatment of inflammatory pain 27, 28, 29. Furthermore, TRPV4 is regarded as an early biomarker of skin carcinogenesis 30.

A series of previous studies have revealed the gating properties and distribution of the thermosensitive proteins in various organs and their participation in many physiological functions as well as their involvement in many pathological processes in the human body. The esophageal epithelium is frequently exposed to stimuli (such as thermal, mechanical, and/or hypotonic) that will activate thermo‐TRPVs, while the expression and function of thermo‐TRPVs in the human esophagus are not yet defined. In this study, we probed for the expression of thermo‐TRPVs in one nontumor esophageal squamous cell line and two esophageal squamous cell carcinoma cell lines, and to our knowledge, for the first time, we characterized the gene expression and cellular localization of TRPV1, TRPV2, and TRPV4 in esophageal squamous cells. Characterization of their functional activities was based on the measurements of Ca^2+^ transients and underlying currents mediated by their selective activations. Their roles in the proliferation and migration of the cell lines were also explored.

## Materials and methods

### Agonists and antagonists

Capsaicin, AMG9810, and ruthenium red were obtained from Sigma‐Aldrich (St. Louis, MO, USA); O1821 and tranilast were purchased from Cayman (Ann Arbor, Michigan, USA) and TargetMol Chemical (Boston, MA, USA), respectively. The chemicals were dissolved in DMSO (the maximal final concentration of DMSO was never exceeded 0.1% throughout the study) and diluted in PBS or extracellular solutions (pH 7.4) to obtain the desired concentrations. Agonists and antagonists were used at the concentrations based on our pre‐experiments on ESCC cells and referred to the EC_50_ or IC_50_ as recommended by the suppliers (Table 1). Matching volumes of DMSO were used as controls.

**Table 1:** Specificity of agonists and antagonists. Data show the compounds’ EC_50_ and IC_50_ where available. A vehicle control (0.1% DMSO) was used where appropriate

Agonist/Antagonist	Receptor	EC_50_	IC_50_	Supplier
Capsaicin	TRPV1	15.2 μm		Sigma
AMG9810	TRPV1		17 nm	Sigma
O1821	TRPV2	25 μm		Cayman
Tranilast	TRPV2		69 μm	TargetMol
Ruthenium red	TRPV1–4		45 μm	Sigma

### Cell culture

The normal esophageal squamous cell line NE2 (kindly provided by Prof. GSW Tsao, Hong Kong University) was immortalized by expression of human telomerase reverse transcriptase (hTERT) and retains nontumorigenic characteristics 31, 32. NE2 cells (passages 6–11) were cultured in a 1 : 1 ratio of Defined Keratinocyte‐SFM (DKSFM) supplemented with growth factors (Gibco, Cat#: 10784‐015) and Epilife medium supplemented with Epilife Defined Growth Supplement (EDGS) growth factors (Gibco, Cat#: S‐012‐5). The human esophageal squamous cell carcinoma (ESCC) cell lines Eca109 (Cat#: TCHu 69) and TE‐1 (Cat#: TCHu 89) were purchased from the Cell Bank of Chinese Academy of Sciences (Shanghai, China). Both ESCC cell lines have been used widely in ESCC‐related studies over decades 33, 34, 35. The ESCC cells (passages 9–17) were cultured in RPMI 1640 medium (Invitrogen, Carlsbad, CA, USA*)* supplemented with 1 mm L‐glutamine and 10% fetal bovine serum (Gibco, Waltham, Massachusetts, USA, Cat#: 11875093). Cells were cultured in a humidified incubator with 5% CO_2_ at 37 °C. The medium was replaced every 3 days, and the cells were subcultured when they reached 85% confluence.

### Thermal stimulation protocol

For proliferation and migration assays, cells cultured in 6‐well plates were exposed to heat stimulation in a water bath thermostat (Sanli Instruments, Shenzhen, China). The water bath temperature (*T*) was set to 5–7 °C higher than each *T*_tested_ for quick thermal conduction through the base of the culture plate. The water bath temperature was automatically maintained by a thermostat. A plastic holder that fit a 6‐well plate was placed into the water in the thermostat, then the medium was pipetted away, and the plates were positioned on the holder and immersed approximately 6 millimeters in the water. Temperatures of the inner surface of the plates (with cells) were monitored by an infrared thermometer (Wahome, Zhongshan, Guangdong, China). Cells were exposed to heat stimulation for the indicated time course, and in most cases, desired temperatures were obtained within 30 s. The detail of thermal stimulation protocols for Ca^2+^ imaging and patch‐clamp experiments will be shown in *Intracellular Calcium Imaging* and *Electrophysiology*.

### Total RNA extraction

Total RNA was extracted from each 6‐well culture plate using HiPure Total RNA Kits (Mage Biotech, Guangzhou, China). Briefly, the contents of each well were trypsinized, collected, and homogenized in 350 μL of RL Buffer/β‐ME, and the cell lysate was transferred to a gDNA Filter Micro Column and centrifuged. The filtrate was then mixed with an equal volume of 70% ethanol, and the mixture was centrifuged in a HiPure RNA Micro Column. Subsequently, the column was washed twice at 8000 ***g*** using 600 μL of Buffer RW for each wash; thereafter, 50 μL of RNase‐free water was added to dissolve the RNA extracted above, and the column was centrifuged at 13 000 ***g*** to collect the filtrate‐containing total RNA. RNA quantity and quality were measured by NanoDrop ND‐1000. RNA samples were kept at −80 °C for future use.

### Reverse‐transcription PCR

The reverse‐transcription mixture included 2 μL of PrimeScript Two‐Step Enzyme Mix (Takara, Tokyo, Japan), 15 μL of 2 × 1 Step Buffer (Dye Plus), 1 μL of forward primer (100 μm), 1 μL of reverse primer (100 μm), 3 μL of random primers at 100 μm (Takara), 1 μL (500 ng) of total RNA, and 7 μL of RNase‐free ddH2O in a final volume of 30 μL. The mixture was incubated at 72 °C for 15 min and 98 °C for 5 s in a 7279 Thermocycler (Applied Biosystems, Foster City, CA, USA).

### Amplified PCR

A series of PCR primers specific to the TRPV family (TRPV1–4, Table 2) was constructed based upon the published work of Somogyi *et al*. 36, 37 or designed using the NCBI primer tool. Reverse‐transcribed samples were sent out for PCR and electrophoresed on 1.2% agarose gel to check the results of reverse‐transcription PCR in Eca109, TE‐1, and NE2 cells. Thereafter, the products generated from the PCRs were sequenced to determine whether the primers were amplifying the appropriate target. Samples that had not undergone reverse transcription were subjected to PCR as negative controls to ascertain that there was no genomic DNA contamination. 18S RNA primers were served as positive controls.

**Table 2:** Primers used for the amplification of first‐strand cDNA of TRPV1–4. Sequences of human TRPV primer used in the study were designed from NCBI primer designing tool or based on previous work. 18S rRNA was included as a transcript control

Gene	Sequence	Product size, bp
TRPV1		
Forward	5′‐CTCACGAGGAAGGTGAGCTG‐3′	216
Reverse	5′‐TCGATGGCGATGTGCAGTGC‐3′	
TRPV2		
Forward	5′‐CGCCATTGAGAAGAGGAGTC‐3′	378
Reverse	5′‐GCTTACCACATCCCACTGCT‐3′	
TRPV3		
Forward	5′‐GCGTGGAGGAGTTGGTAGAG‐3′	276
Reverse	5′‐CTCTGTGTACTCGGCGTTGA‐3′	
TRPV4		
Forward	5′‐ATCGTCTCAGCAGCCCTCTA‐3′	392
Reverse	5′‐TCGGAAAAGGTCCTTGAAGA‐3′	
18S rRNA		
Forward	5′‐GCCGTTCTTAGTTGGTGGAG‐3′	312
Reverse	5′‐GGACTTAATCAACGCAAGC‐3′	

### Protein extraction and western blotting

Cells were cultured in a 6‐well plate. Firstly, medium was discarded and cells were washed by cold PBS on ice, and then, cells were lysed with a buffer containing Tris/HCl (50 mm), NaCl (150 mm), NaN3 (0.02%), Nonidet P‐40 (1%), SDS (0.1%), sodium deoxycholate (0.5%), leupeptin (0.5 mg·mL^−1^), 500 μm phenylmethylsulfonyl fluoride, and aprotinin (1 μg·mL^−1^). The cell lysate was centrifuged at 13 000 ***g*** for 20 min at 4 °C. After this, the supernatant was carefully collected for western blotting. Protein concentration was determined with BCA kit (Genstar, Beijing, China). The proteins were separated by SDS/PAGE and transferred to nitrocellulose membranes (Pierce, Waltham, Massachusetts, USA), which were blocked at room temperature (24 °C to 26 °C) for 1 h in 5% nonfat milk solution. The membranes were incubated at 4 °C overnight with primary rabbit anti‐human TRPV1 (1 : 300, Alomone, Jerusalem, Israel, Cat#: ACC‐030), TRPV2 (1 : 500; Santa Cruz, CA, USA, Cat#: SC‐22520), TRPV3 (1 : 300, Alomone, Cat#: ACC‐033), and TRPV4 (1 : 500; Santa Cruz, CA, USA, Cat#: SC‐98592) antibodies, rabbit anti‐β‐actin antibody (1 : 2000; CST, Danvers, MA, USA, Cat#: 5125S), then washed by a solution containing (in mm) 130 NaCl, 2.5 KCl, 10 Na2HPO4, 1.5 KH2PO4, 0.1% Tween‐20, and incubated with the horseradish peroxidase‐linked secondary antibodies (goat anti‐rabbit IgG, Beyotime, Nanjing, China) in 5% BSA (pH 7.4) for 2 h at room temperature. Final detection was accomplished with western blot luminol reagents (Thermo Scientific, Waltham, MA, USA). Densitometric quantification of TRPV‐1, 2, 3, and 4 proteins was carried out by using imagej (Bethesda, Maryland, USA).

### Immunofluorescence analysis and microscopy

For immunocytochemical analysis, cells were seeded on coverslips in a large Petri dish overnight for cell attachment, and then, the coverslips were washed with cold PBS 3 min each time for 3 times to discard the debris of cells and medium, and fixed cells on the coverslips with 4% paraformaldehyde for 15 min. After fixation, the cells were washed by PBS 3 min each time for 3 times and were permeabilized with 0.1% Triton X‐100 in PBS for 20 min; thereafter, cells were washed by PBS 3 min each time for 3 times. Subsequently, the cells were blocked with 3% BSA for 1 h at room temperature. The primary antibodies were used at 1 : 200 dilution in 3% BSA. For Eca109 cell staining, rabbit anti‐human TRPV1 primary antibody (1 : 200, Alomone, Cat#: ACC‐030) and Alexa Fluor 594‐conjugated secondary antibody (1 : 1000, Abcam (Cambridge, UK), Cat#: ab206371), mouse anti‐human TRPV2 primary antibody (1 : 200, Alomone, Cat #: ACC‐039) and Alexa Fluor 488‐conjugated secondary antibody (1 : 1000, Abcam, Cat#: ab150113), rabbit anti‐human TRPV4 primary antibody (1 : 200, Alomone, Cat#: ACC‐034), and Alexa Fluor 594‐conjugated secondary antibody (1 : 1000, Abcam, Cat#: ab206371) were used. For NE2 cells, rabbit anti‐human TRPV1 primary antibody (1 : 200, Alomone, Cat#: ACC‐030), mouse anti‐human TRPV2 (1 : 1000, Abcam, Cat#: ab206371), rabbit anti‐human TRPV4 primary antibodies (1 : 200, Alomone, Cat#: ACC‐034), and Alexa Fluor 555‐conjugated secondary antibody (1 : 1000, Abcam, Cat#: ab150070) were used. All primary antibodies were incubated overnight at 4 °C and then washed 3 min each time for 3 times in PBST (PBS supplemented with 0.1% Tween‐20). The cells were incubated with secondary antibodies (1 : 1000 dilution in 3% BSA) at room temperature for 1 h and then washed 3 min each time for 3 times with PBST. All cells were incubated with DAPI for 6 min to stain the nucleus then washed 3 min each time for 3 times by PBST. Each coverslip was mounted onto 10 μL of antifading solution on a glass slide. All images were taken on a confocal laser scanning microscope (LSM‐710, Zeiss) and analyzed with the zeiss lsm (Oberkochen, Germany) image examiner software and Adobe Photoshop.

### Intracellular calcium imaging

Cells were cultured in 3‐cm‐diameter glass‐bottom dishes for 24 h; thereafter, medium was discarded and dishes were washed three times using 4 °C Hank's balanced salt solution (HBSS), and then, cells were pre‐incubated with 5 μm Fura‐2 AM (Dojindo Laboratories, Kumamoto, Japan) in 1 mL HBSS [0.05% Pluronic F‐127 (Dojindo Laboratories, Kumamoto, Japan) was included to facilitate Fura‐2 AM to transport into the cells] in the dishes for 45 min at 37 °C in dark. Subsequently, the pre‐incubated solution was pipetted away and cells were washed three times with HBSS to eliminate the extracellular Fura‐2 AM, and then, 1 mL of HBSS was added and cells were incubated at 37 °C in dark for 20 min for the full de‐esterification of intracellular Fura‐2 AM. The dishes were mounted on the stage of an inverted microscope (Eclipse Ti‐U, Nikon, Shinagawa, Tokyo, Japan). Unless indicated otherwise, Fura‐2 AM fluorescence was measured at room temperature (24–25 °C) using a digital imaging system (MetaFluor software, Molecular Devices, Sunnyvale, CA, USA) and alternately exposed to excitation wavelengths of 340 and 380 nm. The ratio (F340/380) refers to a relative index of changes in [Ca^2+^]_i_. The field of interest contained 25–50 fluorescent cells. Results were plotted as a mean ratio of F340/380 nm ± SEM, and *n* values indicate the number of experiments per data point. The measurements lasted between 6 and 14 min. During the first 0.5–3 min, [Ca^2+^]_i_ baseline levels were measured. A control test using the control dish was run through the equal time course of each Ca^2+^ imaging measurement. Some TRPV channel activators and inhibitors were dissolved in a stock solvent dimethyl sulfoxide (DMSO) and further diluted in HBSS to obtain the desired working solutions. The DMSO concentration did not exceed 0.1% which would not affect [Ca^2+^]_i_ (data not shown). Drug administration and washout were conducted manually during the experiments. For thermal stimulation, HBSS was heated via a water bath in Eppendorf tubes in a mini thermostat (TZ, Suzhou, Jiangsu, China) to the desired temperature and the heated HBSS was applied to cells manually, and then, the HBSS temperature was monitored by an infrared thermometer (Wahome, Zhongshan, Guangdong, China).

### Electrophysiology

Eca109 cells, which were primarily dispersed by 0.05% trypsin with 0.2 mg·mL^−1^ EDTA for less than 60 s and then were allowed to adhere to glass coverslips (CitoGlas, Haimen, Jiangsu, China), were utilized in the whole‐cell patch‐clamp analysis within 4 h after the treatment. Patch‐clamp recordings were carried out on the stage of an inverted microscope (TI‐S, Nikon, Shinagawa, Tokyo, Japan) at 24–25 °C unless noted otherwise. Glass coverslips with adherent cells were mounted to a small perfusion chamber with the following extracellular solution (in mm)**:** 135 NaCl, 5 KCl, 2 CaCl_2_, 1 MgCl_2_, 10 glucose, and 10 HEPES, with pH adjusted to 7.4 using NaOH. Patch pipettes made of borosilicate glass (Boxin, Beijing, China) were pulled in a micropipette puller (P‐97, Sutter Instrument, Novato, CA, USA) displayed resistances of 3.5 to 5.5 Ω when filled with the intracellular solution (in mm): 144 KCl, 2 MgCl_2_, 10 HEPES, and 5 EGTA. The pH was adjusted to 7.2 with KOH. Currents were recorded in the whole‐cell patch‐clamp configuration using an Axopatch 200B amplifier controlled by a Digidata 1440 and pclamp 10.2 software (Molecular Devices, Sunnyvale, CA, USA). Recording data were filtered at 1 kHz and sampled at 5–10 kHz. Series resistance (*R*_s_) was compensated to 75%. Whole‐cell capacitance was recorded from the amplifier settings. Data were rejected when *R*_s_ changed >20% or leak currents were >50pA during recording. TRPV1 currents were activated with 100‐ms pulse step from −80 to +100 mV in increments of 20 mV (*V*_h_ = −60 mV). A voltage step protocol consisting of 100‐ms depolarizing pulses from −100 to +100 mV in steps of 20 mV with 5 s of time interval, from a *V*_h_ of −60 mV, was used for heat‐activated TRPV1. Heat (44 °C) stimulation and temperature ramps (0.5–1 °C·s^−1^) from 25 to 35 °C were generated by heating the bath solution via an automation temperature controlling heater (TC‐324B, Warner Instruments, Hamden, CT, USA). For TRPV4, voltage ramps (200 ms) from −100 mV to +100 mV were applied every 5 s from a holding potential of 0 mV. Data were analyzed and displayed with Origin 8.6 (OriginLab, Northampton, MA, USA) or Clampfit 10.2 (Axon Instruments, Union City, CA, USA). Drugs were applied to cells by using a rapid solution changer (RSC‐200, Science Instruments).

### Cell proliferation assay

Cells were pretreated in three ways: added with indicated dose of thermo‐TRPV activators and inhibitors (dissolved and remained in culture medium, until next medium renewal) or exposed to 44 °C heat stimulation (water bath, three times per day, 1 min per time for brief treatment or once per day, 5 min per time for prolonged treatment) or exposed to hypotonic medium (220 m Osm, three times per day, 1 min per time for brief treatment and once per day, 5 min per time for prolonged treatment) for up to 12 days. Heat stimulation was performed via a water bath in a thermostat monitoring with an infrared thermometer (Wahome, Zhongshan, Guangdong, China). The Cell Counting Kit‐8 (CCK‐8; Dojindo Laboratories, Kumamoto, Japan) was used as a colorimetric assay to assess the rate of cell proliferation. Briefly, cells (5 × 10^3^ cells/well) were seeded into 96‐well plates with 100 μL of culture medium for each well. Each sample had five replicates. At the indicated time points, the medium was replaced by 100 μL fresh culture medium; an equal volume of cell‐free culture medium was added to each well in the same plate served as the blank group. Subsequently, 10 μL CCK‐8 solution was added to each well. Plates were incubated for 3–4 h at 37 °C before absorbance measurement at 450 nm using a multimode microplate reader (LB943, Berthold, Bad Wildbad, Germany). The percentage of viable cells was calculated according to a previously published report 38: % Proliferative rate = (OD_treated_ − OD_blank_)/(OD_control_ − OD_blank_) × 100%. The growth curves were constructed based on the OD values.

### Cell wound healing assay

Two parallel lines were drawn on back of the 6‐well plates with a marker pen before cell seeding. Cells were pretreated with indicated dose of thermo‐TRPV activators and inhibitors (dissolved and remained in culture medium, until next medium renewal) or exposed to 44 °C heat stimulation (water bath, three times per day, 1 min per time) or to hypotonic medium (220 m Osm, three times per day, 1 min per) for up to 12 days for Eca109 cells (up to 17 days for NE2 cells). Heat stimulation was performed via a water bath in a thermostat monitoring with an infrared thermometer (Wahome, Zhongshan, Guangdong, China). On the following day, the culture medium was removed and replaced with 0.5% FBS/RPMI 1640 medium. Twenty‐four hours later, a straight wound line that was perpendicular to those parallel lines was drawn across the attached and almost 100% confluent cell layer with yellow pipette tips. Therefore, the wound gap between two parallel lines was marked. Pictures of the marked gap were taken at 24 h and 48 h after the gaps were created. The wound gaps (in mm) were quantified by using image‐pro plus software and calculated by the equation: % migration distance = (gap_0 h treated−_gap_24 h or 48 h treated_)/(gap_0 h control−_gap_24 h or 48 h control_) _*_100% 39.

### Statistical analysis

Data from at least three independent experiments were subjected to analysis by spss software (SPSS Inc., Chicago, IL, USA) and are shown as the mean ± standard error of the mean (SEM). Normal distribution was assessed prior to performing parametric analysis. When appropriate, a paired‐samples or unpaired‐samples *t*‐test was used to analyze differences between experimental and control groups. The data of calcium imaging were analyzed by one‐way ANOVA, followed by Fisher's least significant difference (LSD) test for multiple comparisons, and migration experiments were analyzed by two‐way ANOVA. *P* < 0.05 was considered as statistically significant.

## Results

### Expression of thermo‐TRPVs

**Figure 1:**
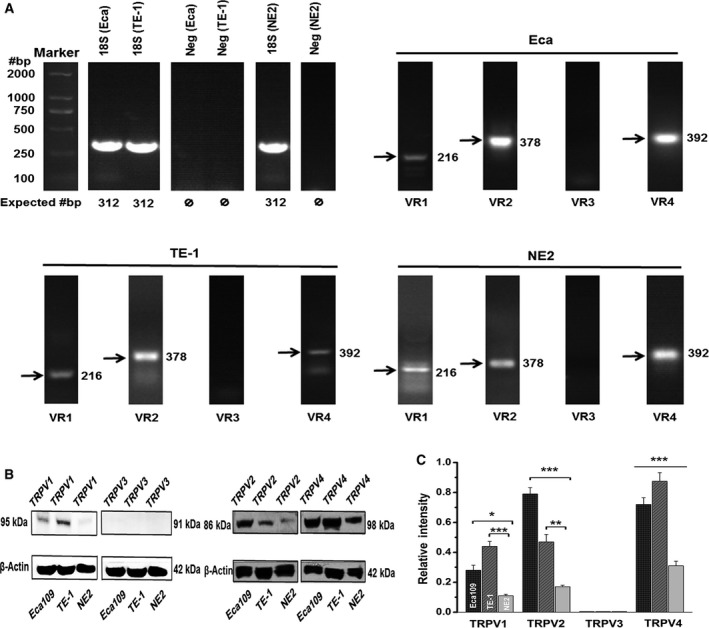
The mRNA and protein expression of thermo‐TRPVs in nontumor esophageal squamous and ESCC cell lines. (A) mRNA of TRPV1, TRPV2, and TRPV4 were detectable in all three cell lines, whereas TRPV3 was absent. (B) Western blot bands for TRPV1 (95 kDa), TRPV2 (86 kDa) and TRPV4 (98 kDa) were present in Eca109, TE‐1, and NE2 cells. β‐actin (42 kDa) staining was used to confirm that an equal amount of protein was loaded in each lane and normalize the densitometric results (in C). (C) Densitometric quantification of TRPV‐1, 2, 3, and 4 protein among three cell lines. Experiments were conducted at least in triplicate. Data were relative to β‐actin and represent the mean ± SEM of the indicated TRPV relative protein expression. Eca, Eca109; VR, TRPV; Neg, negative; *P < 0.05, **P < 0.01, ***P < 0.001.

To investigate gene expression of thermo‐TRPVs among the cell lines, reverse‐transcription PCR (RT‐PCR) was carried out. Total RNA was extracted from Eca109, TE‐1, and NE2 cells, respectively. The total RNA was subsequently purified and subjected to reverse‐transcription PCR to obtain first‐strand cDNA; thereafter, specific primers for TRPV1–4 (Table 2) were used to amplify the segments of interest. TRPV‐1, 2, and 4 were found to be expressed in all 3 cell lines, whereas TRPV3 could not be detected in any of the cell lines, indicating that the TRPV‐1, 2, and 4 genes were all transcribed into mRNA in all 3 cell lines while TRPV3 was not transcribed. The products derived from the PCRs were sequenced to verify that the primers were amplifying the appropriate target (data not shown). 18S rRNA gene was selected as a positive control and strongly expressed in all cell lines. Total RNA extracted from Eca109, TE‐1, and NE2 but not subjected to reverse transcription was loaded as negative controls. No amplification product was observed in the negative control, therefore excluding the possibility of nonspecific amplification in our experiments (Fig. 1A).

Western blots were conducted to explore the protein expression of thermo‐TRPVs in the cell lines; as shown in Fig. 1B, TRPV‐1, 2, and 4 were all found to be expressed in the Eca109, TE‐1, and NE2 cells. While TRPV3 was not detectable in any of these 3 cell lines, which was consistent with the result of the mRNA experiments (Fig. 1A), it should be noted that TRPV‐1, 2, and 4 were found to be upregulated among the ESCC cells compared with the nontumor esophageal squamous cells (Fig. 1B,C), implying that they may involve in the pathology of ESCC.

### Localization of thermo‐TRPVs

**Figure 2:**
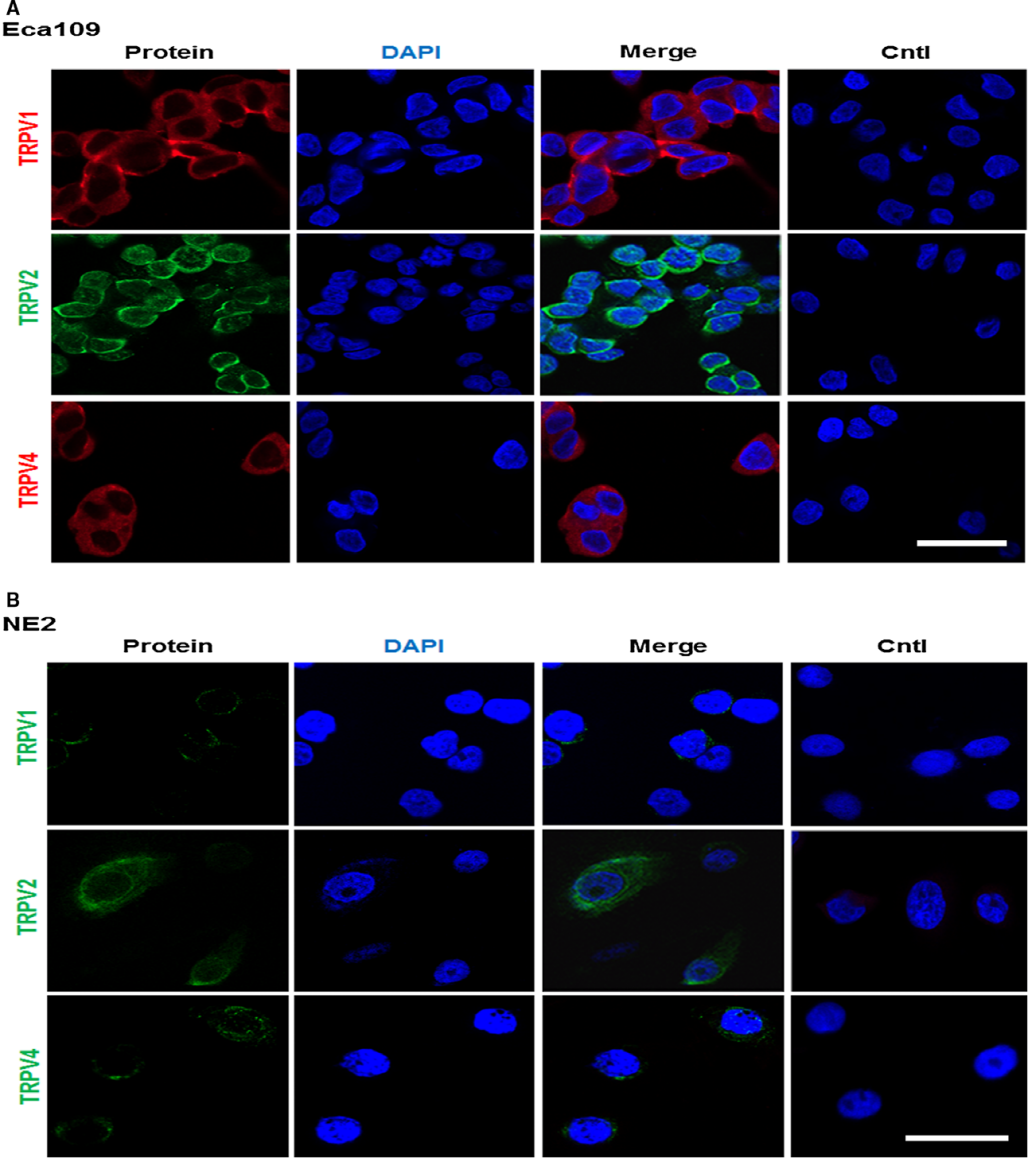
Localization of thermo‐TRPVs in nontumor esophageal squamous and ESCC cell lines. (A) The staining demonstrated that TRPV1 was expressed and mainly located in the plasma membrane of Eca109 (in red). TRPV‐2 and 4 were also found to be expressed and predominantly resided in the plasma membrane of Eca109 cells (TRPV2 in green; TRPV4 in red). DAPI was used to stain the DNA in the nucleus in all experiments (in blue). Eca109 cells which were omissive of primary antibody during the staining procedure were used as negative controls. (B) It showed that TRPV‐1, 2, and 4 were all expressed and mainly resided in the plasma membrane of NE2 (in green). NE2 cells which were omissive of primary antibody during the staining procedure were used as negative controls. Cntl: control. Bar = 10 μm.

Immunocytofluorescence was performed to investigate the expression and localization of thermo‐TRPVs in nontumor esophageal squamous and ESCC cell lines. As demonstrated in Fig. 2A, TRPV‐1, 2, and 4 were found to be expressed and mainly localized to the plasma membrane of the Eca109 cells. Of note, TRPV2 was also found to be partly present in the cytoplasm of Eca109 cells. For the NE2 cells, as illustrated in Fig. 2B, TRPV‐1, 2, and 4 were all found to be expressed and predominantly resided in the plasma membrane. Together, these findings suggest that TRPV‐1, 2, and 4 were all expressed among the cell lines which was in accordance with the results of RT‐PCR and western blot (Fig. 1A,B). Additionally, traffic of TRPV2 might be modulated and it may function intracellularly in the ESCC cells. DAPI was used to stain the DNA in the nucleus and allow easy visualization of the nucleus under the microscope 40. Cells which were omissive of primary antibody during the staining procedure were used as negative controls; no staining but DAPI was observed among the negative controls, which excluded the possibility of nonspecific staining in the cells (Fig. 2A,B).

### Functional analyses of thermo‐TRPVs in ESCC cells via calcium imaging assay

**Figure 3:**
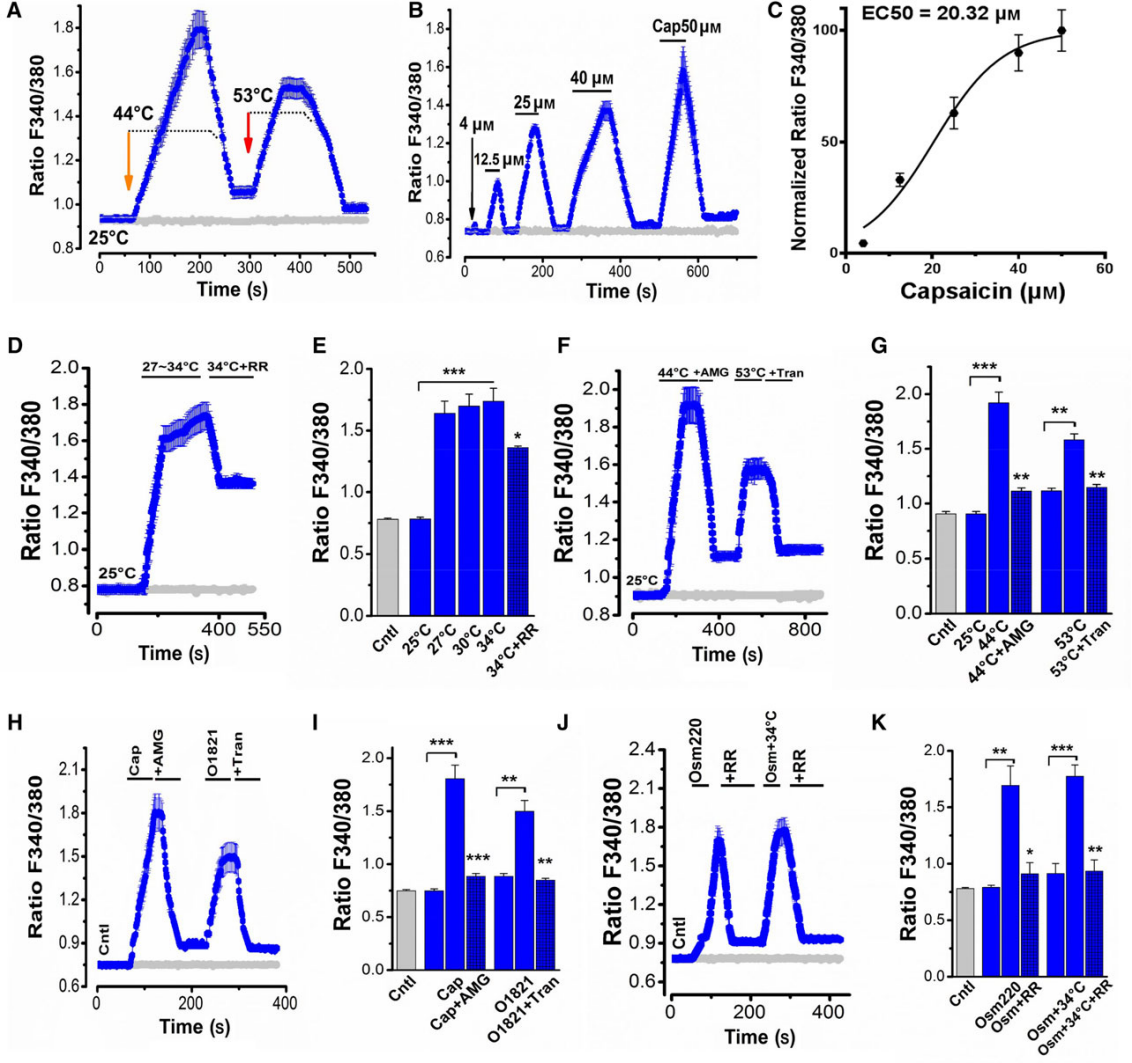
Activation of thermo‐TRPVs by different temperature ranges and agonists resulting in the elevation of intracellular calcium in Eca109 cells. Washout was conducted between every two drug applications by using HBSS at room temperature. (A) Intracellular calcium ([Ca^2+^]_i_) (ratios of F340/380) were increased in response to 44 **°**C and 53 °C HBSS incubation (*n* = 25–39 cells). Gray curve shows the basal ratio of F340/380 in the control group. (B) Increases in [Ca^2+^]_i_ were evoked by the application of capsaicin in a dose‐dependent manner (*n* = 30–39 cells). (C) Dose–response relationship of capsaicin for inducing elevation of [Ca^2+^]_i_ which is shown in B. The curve was fitted with a Hill equation to obtain an EC_50 _= 20.32 μm and *n*_H_ = 1.72 for capsaicin. (D) [Ca^2+^]_i_ was enhanced gradually but significantly in response to a ramp heat (27–34 **°**C) stimulation and inhibited markedly by simultaneous application of 15 μm ruthenium red (RR) (*n* = 35–50 cells). (E) Summary of [Ca^2+^]_i_ oscillation shown in D. (F) [Ca^2+^]_i_ was elevated considerably on the exposure to 44 **°**C and 53 °C and suppressed by AMG9810 (10 nm) and tranilast (100 μm), respectively (*n* = 35–45 cells). AMG9810 is a TRPV1 inhibitor; tranilast is a TRPV2 inhibitor. (G) Summary of [Ca^2+^]_i_ mobilization shown in F. (H) [Ca^2+^]_i_ was enhanced profoundly in the presence of 20 μm capsaicin and inhibited by the co‐administration with AMG9810 (10 nm); [Ca^2+^]_i_ was increased significantly in the presence of O1821 (30 μm), a TRPV2 activator, and suppressed substantially by the co‐application of tranilast (100 μm) (*n* = 30–45 cells). (I) Summary of [Ca^2+^]_i_ mobilization shown in H. (J) [Ca^2+^]_i_ was enhanced markedly on the exposure to the hypotonic HBSS (220 m Osm) and inhibited significantly by the co‐application of ruthenium red (RR, 15 μm); heat stimulation (34 **°**C) potentiated the hypotonic effect, and the overall effect was abrogated by RR (15 μm) (*n* = 33–45 cells). (K) Summary of [Ca^2+^]_i_ mobilization shown in J. Cntl, Control; Cap, capsaicin; RR, ruthenium red; AMG, AMG9810; Tran, tranilast; Osm220, osmotic pressure 220 mm Hg. *P < 0.05, **P < 0.01, ***P < 0.001.

It has been suggested that thermo‐TRPVs are nonselective for cations and all permeable to Ca^2+^ 41; activation of thermo‐TRPVs will induce the inward currents of multiple cations including Na^+^ and especially Ca^2+^ 4, 5. To examine whether the expressed thermo‐TRPVs are functional in our experimental cell lines, we conducted Ca^2+^ imaging assay on Eca109 and NE2 cells in which Fura‐2 AM was used as a probe and intracellular calcium ([Ca_2_^+^]_i_) was determined by fluorescent ratio of 340/380 nm (ratio F340/380). Data shown in Fig. 3 were derived from experiments performed on Eca109 cells. The cellular ratio F340/380 was increased by exposing the cells to sequential heat stimulation (44 **°**C and 53 **°**C, which is the putative activation temperature threshold for TRPV‐1 and 2, respectively) 3, 6. To minimize the influence by a temperature‐dependent spectral shift of the fluorochrome, the Fura‐2‐charged cells were treated with 10 μm ionomycin (Sigma‐Aldrich) for 10 min prior to 44 **°**C and 53 **°**C exposures. As illustrated in Fig. 3A, the ratio amplitude in response to the stimulation of 44 **°**C was increased and was higher than that of 53 **°**C; however, the latter remained at a relatively sustained stable level, it could be explained by different channel kinetics, and it indicated the activation of different ion channels, presumably TRPV‐1 and 2. We then tested the responsiveness of the cells to a TRPV1‐specific agonist, capsaicin. Capsaicin was applied onto the cells from low to high doses (4, 12.5, 25, 40, and 50 μm) and with a washout (using HBSS) interval between every two applications to avoid the tachyphylaxis to capsaicin. As shown in Fig. 3B, ratio F340/380 was enhanced by the application of capsaicin in a dose‐dependent manner (capsaicin doses over 50 μm obtained similar effects to that of 50 μm, data not shown). The dose–response relationship curve was fitted by a Hill equation and obtained an EC_50_ of 20.32 μm with an *n*_H_ = 1.72 for capsaicin (Fig. 3C), which indicated apparent positive cooperativity among the capsaicin binding sites which is in agreement with previous reports 4, 22. When the cells were exposed to heat stimuli or heat applications with relevant thermo‐TRPV inhibitors, [Ca_2_^+^]_i_ was mobilized as follows (Fig. 3D–G and Fig. S1A): [Ca_2_^+^]_i_ was continuously significantly increased when the cells were stimulated by a ramp heat stimulation between 27 **°**C˜34 **°**C (*P *<* *0.001 to [Ca_2_^+^]_i control_), while heat stimulation (34 **°**C, within the activation temperature range for TRPV4, which excludes TRPV1 and TRPV2 thermo‐activation) was simultaneously applied with 15 μm ruthenium red (RR, a TRPV's inhibitor), the increase of ratio F340/380 was shortly suppressed dramatically (*P *<* *0.05 to [Ca_2_^+^]_i 34 **°**C_), indicating that the elevation of [Ca_2_^+^]_i_ was mediated by TRPV4, which was consistent with previous studies 19, 42. When the cells were exposed to heat (44 **°**C), a clear rise in [Ca_2_^+^]_i_ (*P *<* *0.001 to [Ca_2_^+^]_i control_) appeared which was inhibited substantially (*P *<* *0.01 to [Ca_2_^+^]_i 44 **°**C_) by the TRPV1 inhibitor AMG9810 (10 nm), suggesting that the rise in [Ca_2_^+^]_i_ was induced by the activation of TRPV1. When the cells were exposed to 53 **°**C stimulation, a substantial increase in [Ca_2_^+^]_i_ (*P *<* *0.01 to [Ca_2_^+^]_i control_) could be observed and that was abolished by the TRPV2 inhibitor tranilast (100 μm) (*P *<* *0.01 to [Ca_2_^+^]_i 53 **°**C_). In addition, as shown in Fig. S1A, the increase in [Ca_2_^+^]_i_ was slightly and not significantly affected by AMG9810 (10 nm) (*P *>* *0.05), indicating that TRPV1 might also be activated on the exposure to 53 **°**C stimulation besides TRPV2, while the increase in [Ca_2_^+^]_i_ was mainly attributed to the activation of TRPV2. To further confirm the functionality of thermo‐TRPVs in ESCC cells, we evaluated the responsiveness of the cells to various thermo‐TRPV activators and inhibitors. As demonstrated in Fig. 3H–K and Fig. S1B, [Ca_2_^+^]_i_ was elevated markedly in response to 20 μm capsaicin (*P *<* *0.001 to [Ca_2_^+^]_i control_) and the elevation was inhibited significantly by AMG9810 (10 nm) (*P *<* *0.001 to [Ca_2_^+^]_i capsaicin_), indicating that the oscillation of [Ca_2_^+^]_i_ was modulated by TRPV1. A substantial rise in [Ca_2_^+^]_i_ (*P *<* *0.01 to [Ca_2_^+^]_i control_) was observed in the presence of O1821 (30 μm), a newly developed TRPV2 activator 43, while the rise in [Ca_2_^+^]_i_ was suppressed significantly by 100 μm tranilast (*P *<* *0.01 to [Ca_2_^+^]_i O1821_), suggesting that the mobilization of [Ca_2_^+^]_i_ was mediated by TRPV2. When the cells were exposed to hypotonic HBSS (220 m Osm), a pronounced increase in [Ca_2_^+^]_i_ (*P *<* *0.01 to [Ca_2_^+^]_i control_) was induced and it was abrogated by 15 μm ruthenium red (*P *<* *0.05 to [Ca_2_^+^]_i Osm220_), when heat (34 **°**C) stimulation and hypotonic HBSS (220 m Osm) were applied simultaneously, the elevated effect in [Ca_2_^+^]_i_ was potentiated (*P *<* *0.001 to [Ca_2_^+^]_i control_), and it was again inhibited considerably by ruthenium red (*P *<* *0.01 to [Ca_2_^+^]_i Osm220+34 **°**C_). However, these effects were neither significantly affected by AMG9810 nor tranilast (*P *>* *0.05 to [Ca_2_^+^]_i Osm220_ and *P *>* *0.05 to [Ca_2_^+^]_i Osm220+34 **°**C_, Fig. S1B). Collectively, these findings further support the notion that the oscillations in [Ca_2_^+^]_i_ in response to hypotonic HBSS (220 m Osm) were mainly modulated by TRPV4. We obtained similar outcomes in another set of experiments that was conducted on NE2 cells, where the amplitudes of calcium mobilization were less than those observed in Eca109 cells (Data not shown). These may due to the lower expression levels of thermo‐TRPVs protein in NE2 cells (Fig. 1B,C).

### Functional analyses of thermo‐TRPVs in ESCC cells via whole‐cell patch‐clamp recording

**Figure 4:**
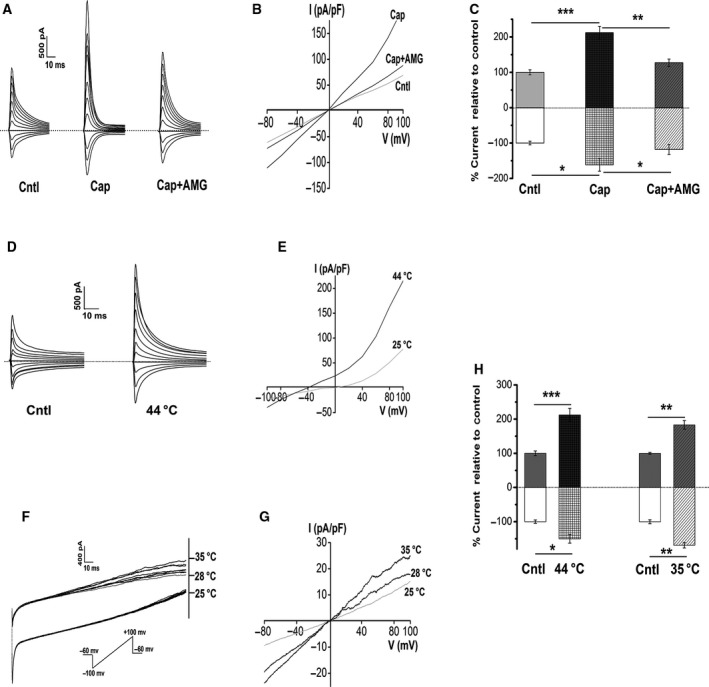
Activation of thermo‐TRPVs in Eca109 cells by different temperature ranges and agonist in a whole‐cell patch‐clamp configuration. (A) Representative membrane currents in response to 20 μm capsaicin in the absence or presence of 10 nm AMG9810 (*n* = 5 cells). *Dashed lines*, zero current or potential level. (B) Current–voltage (I–V) relationship for the currents shown in A. A large outward rectified current was found in the presence of 20 μm capsaicin. (C) Summary of currents shown in A, note that the outward currents (above zero) and inward currents (below zero) were both enhanced substantially in response to 20 μm capsaicin, and both were inhibited markedly by 10 nm AMG9810; data were normalized to the control. (D) Sample membrane currents on the exposure to heat stimulation (44 **°**C extracellular solution) (*n* = 4 cells). *Dashed lines*, zero current or potential level. (E) I–V relationship for heat‐evoked currents, reverse potential was left shifted to −40 mV by heat stimulation, and a large outward rectified current was seen. (F) Representative current traces in response to a ramp heat protocol [exposure to 25 **°**C–35 **°**C (0.5–1 °C·s^−1^) extracellular solution] (*n* = 4 cells). Dashed lines, initial point of the ramp recording. (G) I–V relationship of the exposure to the ramp heat. (H) Summary of currents shown in D and F, inward currents and outward rectified currents were increased pronouncedly by heat (44 **°**C) stimulation; inward currents and outward rectified currents were elevated substantially by 35 **°**C stimulation. Data represent the mean ± SEM of the indicated number of recordings. Cntl, Control; Cap, capsaicin; AMG, AMG9810. *P < 0.05, **P < 0.01, ***P < 0.001.

To further verify the function of thermo‐TRPVs in ESCC cells, we next investigated the electrophysiological activity of thermo‐TRPVs in the Eca109 cells by using the whole‐cell patch‐clamp configuration. As shown in Fig. 4A, inward currents were enhanced significantly in response to 20 μm capsaicin compared to the control (1109.62 ± 59 pA to 687.26 ± 66 pA, *P* <* *0.05) and inhibited markedly by the TRPV1 antagonist, AMG9810 (10 nm) (1109.62 ± 59 pA to 811.16 ± 73 pA, *P *<* *0.05, Fig. 4A,C). Large outward currents were seen in the presence of capsaicin (3112.18 ± 75 pA to 1494.14 ± 54 pA, *P *<* *0.001 compared with the control) and were suppressed by the co‐application of AMG9810 (3112.18 ± 75 pA to 1867.07 ± 92 pA, *P *<* *0.01, Fig. 4A,B,C). The voltage–current relationship curve revealed the rectification characteristic of outward currents induced by capsaicin (Fig. 4B), which is a hallmark for many TRPs 9. The currents induced by capsaicin and inhibited by AMG9810 in our experiments indicated that the transmembrane electrophysiological activity was mediated by TRPV1. A voltage step protocol was applied to further investigate the effect(s) of heat (44 **°**C) exposure on TRPV1. As shown in Fig. 4D‐H, inward current amplitude was increased considerably (from −696.41 ± 25 pA to −1046.14 ± 59 pA, *P *<* *0.05) by the heat (44 **°**C) exposure. Outward rectified currents were also found to be enhanced substantially (from 1126.10 ± 80 to 2389.53 ± 78 pA, *P *<* *0.001) in response to heat (44 **°**C) stimulation. Reverse potential was left shifted from 5 mV (25 **°**C) to −40 mV by heat (44 **°**C) stimulation. Voltage ramps were used to examine the activity of TRPV4. As shown in Fig. 4F‐H, inward currents were increased gradually but significantly on the exposure to the ramp heat stimulation (from 25–35 **°**C, *P *<* *0.01). Outward rectified currents were elevated markedly (from 278.32 ± 41 pA to 436.21 ± 19, pA *P *<* *0.01), and these data indicated but not proved the activation of TRPV4. Due to the unstable condition under higher temperature (> 50 **°**C), we could not record the activity of TRPV2 in response to heat stimulation in our whole‐cell patch‐clamp recordings; however, the activities of TRPV2 could be demonstrated by our calcium imaging experiments (Fig. 4F,H). Together, data derived from our whole‐cell patch‐clamp recordings suggest that the expressed TRPV1 and TRPV4 in the Eca109 cells were activated by capsaicin and/or heat, respectively, and contributed to the membrane currents observed (Fig. 4).

### Recurrent activations of TRPV1 by heat and agonist promoted proliferation of ESCC cells

**Figure 5:**
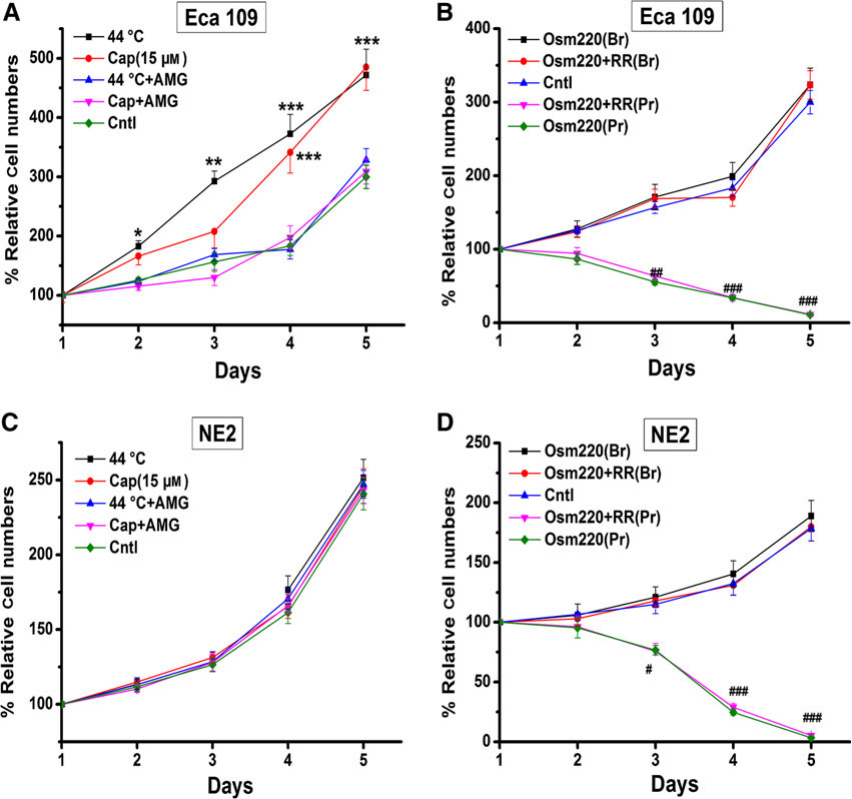
Effects of overactivation of TRPV1 and TRPV4 on the proliferation of Eca109 and NE2 cells. The proliferation curves were constructed based on OD values (for details, see *Methods*). (A) Eca109 cell growth was enhanced significantly by the treatment of 15 μm capsaicin and recurrently brief exposure to heat (44 **°**C); the TRPV1 antagonist AMG9810 (10 nm) could abolish these effects. (B) Eca109 cell proliferation was not affected by recurrently brief exposure to hypotonic solutions (220 m Osm), whereas the prolonged exposure resulted in a large amount of cell death and pronounced decrease in cell numbers. Note that the TRPV antagonist ruthenium red (15 μm) could not reverse the prolonged effect. (C) NE2 cell growth was neither affected by the treatment of 15 μm capsaicin nor by 44 **°**C heat stimulation. (D) NE2 cell proliferation was not affected by recurrently brief exposure to hypotonic solutions (220 m Osm), while prolonged exposure resulted in almost complete cell death. Ruthenium red (15 μm) could not reverse the prolonged effect. Cap: capsaicin; AMG: AMG9810; Osm220: osmotic pressure 220 mm Hg; RR: ruthenium red; Br: brief treatment; Pr: prolonged treatment; Cntl, control. * or *#P* < 0.05, ** or *##P* < 0.01, *** or *###P* < 0.001.

In order to examine the effect of thermo‐TRPVs on the growth of ESCC cells, CCK‐8 assay was performed. Cellular proliferation ability was measured according to the manufacturer's instructions (details in *Methods*). As shown in Fig. 5A, cellular proliferation of Eca109 was enhanced significantly by recurrently brief heat stimulation (*P* < 0.001) and 15 μm capsaicin (*P* < 0.001) (‘overactivation’ was used to describe the condition of recurrent treatments in the current study). Higher dose of capsaicin could result in Eca 109 cell death (data not shown). Meanwhile, the cellular proliferation‐promoting effects by heat stimulation and capsaicin exposure were both inhibited pronouncedly by the TRPV1 antagonist AMG9810 (10 nm) (Fig. 5A), indicating that activations of TRPV1 by heat and capsaicin could promote cellular proliferation of Eca109. In the other experiment, however, cellular proliferation of Eca109 was not affected by the brief treatment of hypotonic medium (220 m Osm) (Fig. 5B), suggesting that the overactivation of TRPV4 has no effect on the proliferation of Eca109 cells. On the other hand, in the extended treatment group, a large amount of Eca109 cell death could be observed and the cell death process could not be reversed by ruthenium red (15 μm) (Fig. 5B), indicating that there was not only the activation of TRPV4, but other mechanisms might also be involved in this process. For the NE2 cells, as was illustrated in Fig. 5C and D, NE2 cell growth was neither affected by the treatment of 15 μm capsaicin nor by 44 **°**C heat stimulation. NE2 cell proliferation was not affected by recurrently brief exposure to hypotonic medium (220 m Osm), while the prolonged exposure resulted in almost complete cell death. Likewise, ruthenium red could not reverse the prolonged effect (Fig. 5D). Together, these data suggested that the ESCC cells were more vulnerable to the overactivation of TRPV1 channels than the nontumor esophageal squamous cells and these effects may be attributed to the higher expression levels of thermo‐TRPVs among ESCC cells (Fig. 1B,C). It is noteworthy that ESCC cells and nontumor esophageal squamous cells were similarly vulnerable to hypotonic stress during the prolonged exposure to hypotonic medium (220 m Osm) (Fig. 5B,D).

### Recurrent activations of TRPV1 and TRPV4 by heat and agonists promoted cellular migration of Eca109

**Figure 6:**
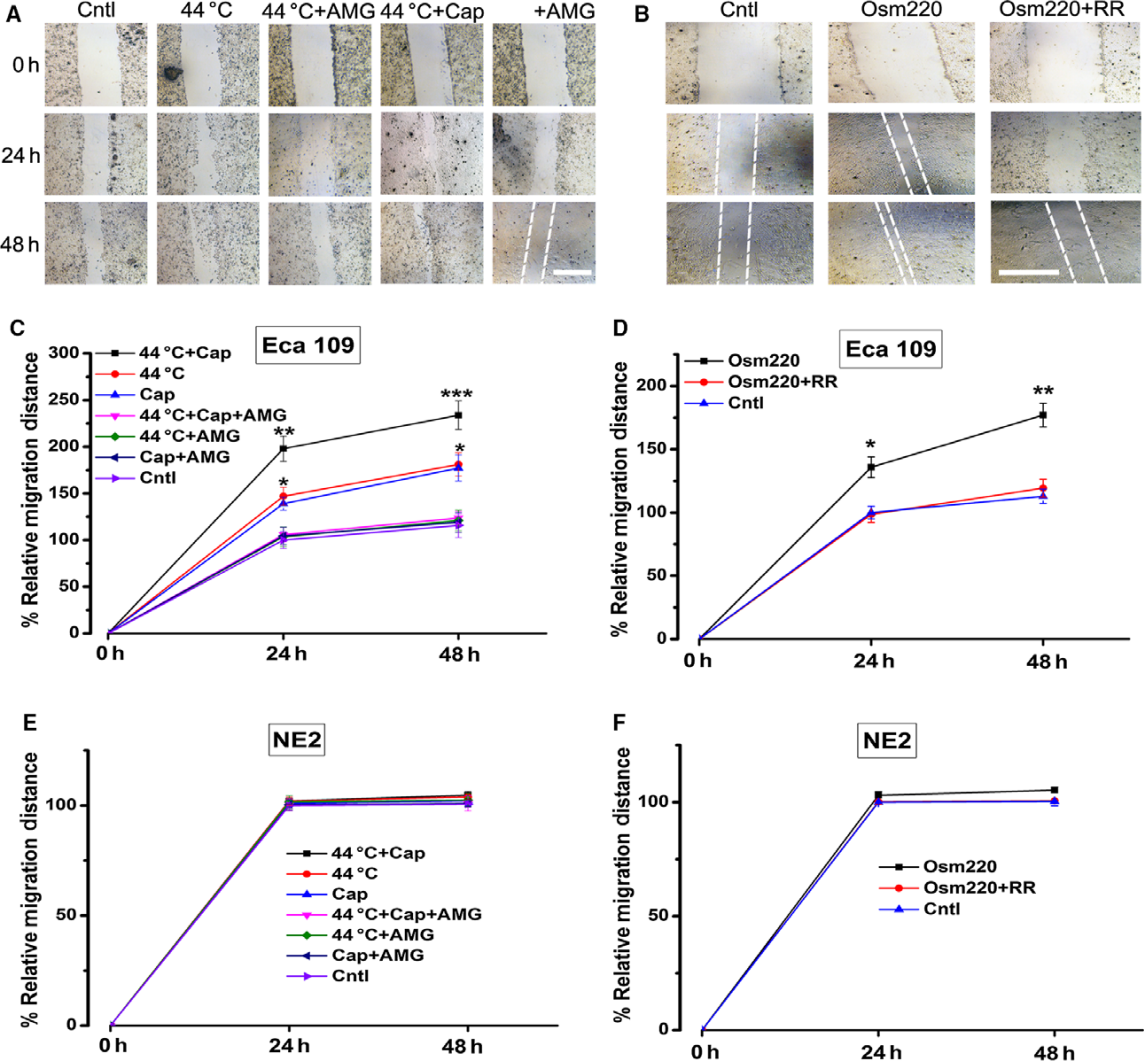
Effects of overactivation of TRPV1 and TRPV4 on the migration of Eca109 and NE2 cells. Cell migration was assessed via a wound healing assay. (A) Representative images of Eca109 cell migration after exposure to capsaicin (15 μm) and/or heat stimulation (44 **°**C water bath). AMG9810 (10 nm) was used as a TRPV1 antagonist. The white broken lines assisted to define the edging of the wounds. (B) Sample pictures of Eca109 cell migration after recurrently brief exposure to hypotonic medium (220 m Osm). Ruthenium red (RR, 15 μm) was used as a TRPV inhibitor. (C) Eca109 cell migration was promoted substantially by the application of 15 μm capsaicin and/or recurrently brief exposure to heat (44 **°**C); cell migration was enhanced much greater by the simultaneous treatment with capsaicin and heat stimuli; these effects could be abrogated by AMG9810 (10 nm). (D) Eca109 cell migration was accelerated considerably by recurrently brief exposure to hypotonic medium (220 m Osm); this effect was compromised by ruthenium red (15 μm). (E) NE2 cell migration was not affected by the application of 15 μm capsaicin and/or heat stimulation (44 **°**C water bath) even up to 17 days. (F) NE2 cell migration was unaffected by recurrently brief exposure to hypotonic medium (220 m Osm) even up to 17 days. Cap, capsaicin; AMG, AMG9810; Osm220, osmotic pressure 220 mm Hg; RR, ruthenium red; Cntl, control. *P < 0.05, **P < 0.01, ***P < 0.001. Bar = 1.0 mm

To assess the effect of activation of thermo‐TRPVs on cellular migration of the ESCC cells, wound healing assay was carried out. As shown in Fig. 6A, C and Fig. S3, the migration velocity of Eca109 cells was markedly enhanced by recurrently brief heat stimulation (44 **°**C) (*P* < 0.05) and 15 μm capsaicin (*P* < 0.05) or the simultaneous application of heat stimulation with capsaicin (*P* < 0.001), respectively; these effects were suppressed significantly by AMG9810 (10 nm) (*P* < 0.05, *P* < 0.001, respectively). In the other assay, Eca109 cell migration was found to be accelerated substantially in the presence of hypotonic medium (220 m Osm) and these effects were abolished by ruthenium red (15 μm) (Fig. 6D). Overall, these data suggested that the overactivation of TRPV1 and TRPV4 significantly promoted cellular migration of the Eca109 cells. For the nontumor esophageal squamous cells, as illustrated in Figs 6E,F and S4, migration of NE2 cells was affected neither by the treatment of 15 μm of capsaicin nor by recurrently brief 44 **°**C heat stimulation even up to 17 days (Fig. S4). Migration of NE2 cells was also unaffected by recurrently brief exposure to hypotonic medium (220 m Osm) even up to 17 days. The migration results suggested that the ESCC cells were more vulnerable to the overactivation of TRPV1 and TRPV4 channels than the nontumor esophageal squamous cells and these effects may result from the higher expression levels of thermo‐TRPVs among ESCC cells (Fig. 1B,C) or different signal pathways exploited by the 2 different types of cells during the activation process.

## Discussion

The esophagus acts as a conduit that transports swallowed food and beverages from the oropharynx to the stomach 44. The esophageal epithelium is easily exposed to various stimuli (including heat) during food ingestion that could activate thermo‐TRPs. Therefore, in this study we focused on the warm sensing‐ or thermal pain‐ related TRPs, namely thermo‐TRPVs. We found that TRPV‐1, 2, and 4 were all expressed at both mRNA and protein levels in the nontumor esophageal squamous cells and esophageal squamous cell carcinoma cells, whereas TRPV3 mRNA transcript and protein were not detectable among all 3 cell lines (Fig. 1A,B). Other groups have reported different expression patterns of thermo‐TRPVs among various organs and tissue cells, such as in the bladder epithelium, vascular smooth muscle cells, chondrogenic cells, and T cells 9, 36, 45, suggesting diverse expression modes and multifunctions of these channels 1, 13, 46. Moreover, we found that the expressed thermo‐TRPVs were all upregulated in the ESCC cells compared with the nontumor esophageal squamous cells, according to the western blot experiments (Fig. 1B,C). Previous studies have suggested that the tumorigenesis process of various types of cancers involves the altered expression of one or more TRP proteins 47, 48. It implied that thermo‐TRPVs might participate in the pathology of ESCC.

Most TRP channels are localized to the plasma membrane, where they have an essential role in the influx of and/or the transcellular machinery that transports Ca^2+^, Mg^2+^, and trace metal ions, but they have also been found to be localized to intracellular membranes 46. Accumulated evidence suggests that intracellularly localized TRP channels actively participate in regulating membrane traffic, signal transduction, and vesicular ion homeostasis 13, 46. Previous work reported that TRPV1 channels localize both to the plasma membrane and to intracellular membranes in human embryonic kidney (HEK) 293 cells 41. However, our findings derived from immunocytofluorescence experiments showed TRPV‐1, 2, and 4 were all expressed and mainly located in the plasma membrane of both ESCC cells (Eca109) and nontumor esophageal squamous cells (NE2) (Fig. 2A,B). Additionally, TRPV2 was found to be partly present in the cytoplasm of ESCC cells. Together, these data demonstrate that the distribution of thermo‐TRPVs is cell type‐dependent and indicates that they may play distinct roles among different cell types.

TRP channels mainly mediate their effects by controlling the concentrations of intracellular calcium ([Ca^2+^]_i_), which acts as a second messenger inside the cells 7. An increase in [Ca^2+^]_i_ in response to stimuli specific to certain TRP channels serves as a good indicator of functional expression for almost all TRP channels (except TRPM4 and TRPM5) as they are Ca^2+^ permeable 41. As shown in Fig. 3, calcium mobilizations were induced by heat stimuli, hypotonic solutions, and various TRPV‐specific activators and were suppressed significantly by corresponding inhibitors, which suggested that the functional activities of the expressed TRPV1, TRPV2, and TRPV4 channels, respectively. It should be noted that the heat‐evoked increase in [Ca^2+^]_i_ sustained longer and not easily returned to the baseline compared with those induced by thermo‐TRPV activators even in the presence of relevant inhibitors, indicating that the cells need more time to manipulate and restore [Ca^2+^]_i_ under the heat stimulation context. Notably, the TRPV2 agonist O1821 (Cayman Chemicals, Ann Arbor, Michigan, USA) is a new synthetic cannabinoid that effectively stimulates TRPV2, but does not stimulate TRPV1 or the cannabinoid receptors 43, 49.

The nonselective currents of thermo‐TRPVs were explored by the whole‐cell patch‐clamp experiments. The step membrane currents increased dramatically when the cells were exposed to 20 μm capsaicin alone and were inhibited markedly by combined application of capsaicin with AMG9810 (10 nm), a potent and selective antagonist of TRPV1 which can significantly antagonize both thermal and capsaicin effects on TRPV1 channels 50 (peak inward currents_cap_ vs. peak inward currents_cap+AMG_, *P *<* *0.05). It is worth noting that in either calcium imaging assays or the patch‐clamp recordings, lower concentration (10 nm) than the IC50 of AMG9810 (17 nm) can effectively antagonize the activation of TRPV1 in Eca 109 cells. The I‐V relationship showed a large outward rectified current induced by 20 μm capsaicin which was inhibited by AMG9810 (peak outward currents_cap_ vs. peak outward currents_cap+AMG_, *P *<* *0.01), suggesting that the transmembrane electrophysiological activities were mediated by TRPV1 (Fig. 4A–C). Step membrane currents including both inward and outward currents were enhanced considerably by the stimulation of 44 °C (peak inward currents_44 °C_ vs. peak inward currents_cntl_ *P* < 0.05, peak outward currents_44 °C_ vs. peak outward current_cntl_ *P* < 0.001). Outward rectification has been regarded as a hallmark characteristic for many TRP channels and was explained due to both reduced single‐channel conductance and open probability at negative potentials 51, 52. It is noteworthy that the reverse potential was left shifted under heat stimulation by 35 mV (Fig. 4E). Vyklicky and Cao had reported the reversal potential for thermally activated TRPV1 currents to be about 0–5 mV in rat's sensory neurons 53, 54. The cause for variation in reverse potential of the heat‐evoked TRPV1 currents in our experiments may be due to different species (human vs. rat) or experimental configurations applied. In the subsequent recordings, the membrane currents were increased substantially in response to the ramp heat stimulation from 25 °C to 35 °C (peak inward currents_35 °C_ vs. peak inward currents_cntl_ *P* < 0.01, peak outward currents_35 °C_ vs. peak outward currents_cntl_ *P* < 0.01) (Fig. 4F–H). Outward rectifications were both seen in heat‐evoked TRPV1 and TRPV4 currents, which are in accordance with previous reports 5, 52. The stimulating temperature range can exclude the activation of both TRPV‐1 and 2, therefore indicated but not proved the currents were mediated by TRPV4 12.

Collectively, data derived from Ca^2+^ imaging and patch‐clamp experiments suggest that the expressed thermo‐TRPVs are functional in the ESCC cells.

Previous studies reported that thermo‐TRPVs were involved in tumorigenesis of various types of cancers 9, 47, 48. Also, TRPV1 and TRPV4 were reported to be involved in modulating cell migration 47, 55. Our previous study suggested that TRPV2 acts as an important enhancer for H_2_O_2_‐induced cytotoxicity in HepG2 cells 56. The fact that upregulation of thermo‐TRPVs in ESCC cells prompted us to test their potential role in the development of ESCC.

Possessing greater abilities in cellular proliferation and migration than normal cells has been regarded as the basic hallmarks of cancerous cells 57, 58. Since the thermo‐TRPVs were found to be upregulated in ESCC cell lines, we next investigated the effects of these ion channels (TRPV1 and TRPV4 in this study) on proliferation and migration ability of ESCC cells. Meanwhile, the nontumor esophageal squamous cell line NE2 was used as a control. As shown in Fig. 5A,B, surprisingly, the cellular proliferation of Eca109 was found to be enhanced significantly in the sustained presence of 15 μm capsaicin. Numerous previous studies documented that capsaicin could cause cellular death of various forms of cancer cells, which rendered the anticancer effects 59, 60. Actually, we also observed similar effects on ESCC cells, but we found that cell death occurred only when ESCC cells were exposed to higher doses of capsaicin (>28 μm, which is above the EC50 for capsaicin to induce increase in [Ca^2+^]_i_, Fig. 3C). Zhang J. H., *et al*. reported that human pancreatic cancer cell growth was inhibited by capsaicin treatment in a dose‐dependent manner with an IC_50_ ~200 μm 61, suggesting that high dose of capsaicin could result in cancer cell death. On the contrary, we found that the proliferation of ESCC cells was promoted substantially by low dose, but in consecutive presence of capsaicin (< 17 μm, which is below the EC50 for capsaicin to induce increase in [Ca^2+^]_i_, (Fig. 3C), indicating that different doses of capsaicin may have distinct effects on the proliferation of cancer cells. Therefore, we propose that the dose of capsaicin should be taken into consideration on the purpose of anticancer effect. Moreover, proliferation of Eca109 cells was promoted markedly by repeatedly brief heat stimulation (44 °C) and this effect was inhibited significantly by AMG9810, which further confirmed that the activation of TRPV1 could promote the proliferation of ESCC cells (Fig. 5A). The proliferation ability was unaffected by the recurrently short‐time treatment with hypotonic medium (220 m Osm), which could activate the channel of TRPV4, suggesting that TRPV4 may not mediate the proliferation of the ESCC cells (Fig. 5B). In contrast to the ESCC cells, proliferation of the nontumor esophageal squamous cells (NE2) was neither affected by capsaicin nor heat stimulation (44 °C) (Fig. 5C), it also remained unaffected on the exposure to hypotonic medium (220 m Osm). The overall data demonstrated distinct response between the tumor cells and the nontumor cells, and this may due to the different expression or activity levels of thermo‐TRPVs between these two types of cells.

Cell migration plays a pivotal role in cancer invasion and metastasis. Many of the components of cellular migration machinery are regulated by the intracellular calcium concentration 47.

The result of migration assay demonstrated that the migration of Eca109 cells was promoted considerably by the overactivation of TRPV1 by 15 μm of capsaicin and/or recurrently brief heat stimulation (44 °C). Although the proliferation of ESCC cells was not affected by the hypotonic stimulation (Fig. 5B), the migration of ESCC cells was accelerated significantly by the hypotonic stress (220 m Osm). With the data in our Ca^2+^ imaging assay, it suggests that the enhanced migration of ESCC cells by hypotonic stimulation was mainly mediated by TRPV4. Previous *in vivo* work reported that sensory neurons did not exhibit osmosensitive inward currents and the activation of peripheral osmoreceptors was abolished by knockout of TRPV4 62, revealing that TRPV4 is the key channel responding to osmotic stimuli, thus further supporting the notion that overactivation of TRPV4 plays a pro‐migration role in ESCC cells.

It is well known that the esophageal epithelium is unavoidably and frequently exposed to thermal, mechanical and/or hypotonic stimulation during food intake; therefore, thermo‐TRPVs are frequently activated which will result in Ca^2+^ entries. Thus, thermo‐TRPVs may play a role in the calcium homeostasis of the esophageal epithelium and the maintenance of its function(s). Our findings in this study show that overactivations of TRPV1 and TRPV4 in the esophageal squamous carcinoma cells by low dose of capsaicin, noxious thermal stimulation and hypotonic stimulation could promote cellular proliferation and/or migration and thus may further promote the development of ESCC.

There are still some limitations for the current study, such as the impact(s) of overactivation of thermo‐TRPVs on the invasive ability, and pro‐angiogenesis capacity in ESCC cells is not explored here. Our ongoing project which is aimed at the detail role(s) of thermo‐TRPVs playing in the carcinogenesis of ESCC will help solve these issues in the near future.

In summary, in this study we found that thermo‐TRPVs were functionally expressed in nontumor esophageal squamous cells and were upregulated in esophageal squamous cell carcinoma cells. Meanwhile, overactivation of TRPV1 and TRPV4 could promote the cellular proliferation and/or migration of ESCC cells. TRPV1 and TRPV4 may play an important role in the development of ESCC.

## Author contributions

ZYL and RQH conceived the original project design. RQH and FW performed and analyzed all experiments. ZXL, SHD, and NC contributed to experimental design with comment on specific experiments from WBM, YL, and YCY RQH drafted the paper along with ZYL, and all authors contributed to the subsequent preparation of the paper and have approved the paper.

## Conflict of interest

The authors declare no conflict of interest.

## Supporting information


**Fig. S1.** Raw western blot data. Uncropped images of western blots in main figure 1B are shown. M: marker; Eca: Eca109; V1: TRPV1; V3: TRPV3.
**Fig. S2.** Activation of specific thermo‐TRPVs in Eca109 cells. (A) [Ca^2+^]_i_ was elevated considerably on the exposure to 53 °C (the activation temperature threshold for TRPV2) and this effect was slightly influenced (*P *>* *0.05) by the co‐administration of AMG9810, a TRPV1 inhibitor. (*n* = 35–40). (B) [Ca^2+^]_i_ was enhanced substantially on the exposure to the hypotonic HBSS **(**220 m Osm); heat stimulation (34 **°**C) potentiated the hypotonic effect and these effects were neither significantly affected by AMG9810 nor Tranilast (*n* = 30–40). (C) A cell was selected for recording by metafluor software during Ca^2+^ imaging measurement, pictures were captured at excitation wavelength 340 nm. Upper panel: control; lower panel: exposure to 53 **°**C HBSS, enhanced intensity of fluorescence_340 nm_ was shown. (D) Representative cell images captured at ratio F340/380 during Ca^2+^ imaging assay, top: control; middle: exposure to 44 **°**C; bottom: exposure to 53 **°**C. Cntl: control; AMG: AMG9810; Tran: Tranilast; Prior: prior to treatment; Osm220: osmotic pressure 220 mm Hg. *ns*: not significant; ***P < 0.001, one‐way ANOVA. Scale bar (in C and D): 10 μm.
**Fig. S3.** Effects of overactivation of TRPV1 by capsaicin on the migration of Eca109 cells. Cell migration was assessed via a wound healing assay. (A) Representative images of Eca109 cell migration after exposure to capsaicin (15 μm) or capsaicin + AMG9810 (10 nm). The white dashed lines assisted to define the edging of the wounds. Scale bar: 1.0 mm.
**Fig. S4.** Impacts of overactivation of TRPV1 and TRPV4 on the migration of NE2 cells. Cell migration was measured via a wound healing assay. (A) Exemplar images of NE2 cell migration after recurrently brief exposure to heat stimuli (44 **°**C water bath) and application of capsaicin (15 μm). AMG9810 was used as a TRPV1 antagonist. (B) Representative pictures of NE2 cell migration after recurrently brief exposure to hypotonic media (220 m Osm). Ruthenium red (RR) was used as a TRPV inhibitor. Cntl: control; Cap: capsaicin; AMG: AMG9810; RR: ruthenium red; Osm220: osmotic pressure 220 mm Hg. Scale bar: 1.0 mm.
