# Supplementary material for: Recurrent activations of transient receptor potential vanilloid‐1 and vanilloid‐4 promote cellular proliferation and migration in esophageal squamous cell carcinoma cells
Source: FEBS Open Bio. 2019 Jan 18;9(2):206–25. doi: 10.1002/2211-5463.12570 (PMC6356177; doi:10.1002/2211-5463.12570)
Supplement: Supplementary file 1 — Fig. S1. Raw western blot data. Uncropped images of western blots in main figure 1B are shown. M: marker; Eca: Eca109; V1: TRPV1; V3: TRPV3. Fig. S2. Activation of specific thermo‐TRPVs in Eca109 cells. (A) [Ca2+]i was elevated considerably on the exposure to 53 °C (the activation temperature threshold for TRPV2) and this effect was slightly influenced (P > 0.05) by the co‐administration of AMG9810, a TRPV1 inhibitor. (n = 35–40). (B) [Ca2+]i was enhanced substantially on the exposure to the hypotonic HBSS (220 m Osm); heat stimulation (34 °C) potentiated the hypotonic effect and these effects were neither significantly affected by AMG9810 nor Tranilast (n = 30–40). (C) A cell was selected for recording by metafluor software during Ca2+ imaging measurement, pictures were captured at excitation wavelength 340 nm. Upper panel: control; lower panel: exposure to 53 °C HBSS, enhanced intensity of fluorescence340 nm was shown. (D) Representative cell images captured at ratio F340/380 during Ca2+ imaging assay, top: control; middle: exposure to 44 °C; bottom: exposure to 53 °C. Cntl: control; AMG: AMG9810; Tran: Tranilast; Prior: prior to treatment; Osm220: osmotic pressure 220 mm Hg. ns: not significant; ***P < 0.001, one‐way ANOVA. Scale bar (in C and D): 10 μm. Fig. S3. Effects of overactivation of TRPV1 by capsaicin on the migration of Eca109 cells. Cell migration was assessed via a wound healing assay. (A) Representative images of Eca109 cell migration after exposure to capsaicin (15 μm) or capsaicin + AMG9810 (10 nm). The white dashed lines assisted to define the edging of the wounds. Scale bar: 1.0 mm. Fig. S4. Impacts of overactivation of TRPV1 and TRPV4 on the migration of NE2 cells. Cell migration was measured via a wound healing assay. (A) Exemplar images of NE2 cell migration after recurrently brief exposure to heat stimuli (44 °C water bath) and application of capsaicin (15 μm). AMG9810 was used as a TRPV1 antagonist. (B) Representative pictures of NE2 cell mi [file FEB4-9-206-s001.docx]

**Supplementary Material**

**Recurrent activations of transient receptor potential vanilloid-1 and -4 promote cellular proliferation and migration in esophageal squamous cell carcinoma cells**

**Rongqi Huang^1,2^, Fei Wang^1^, Yuchen Yang^1^, Wenbo Ma^1^, Zuoxian Lin^1^, Na Cheng^1,^****^3^, Yan Long ^1^ , Sihao Deng^3^ and Zhiyuan Li^1,2,3,4^**

**
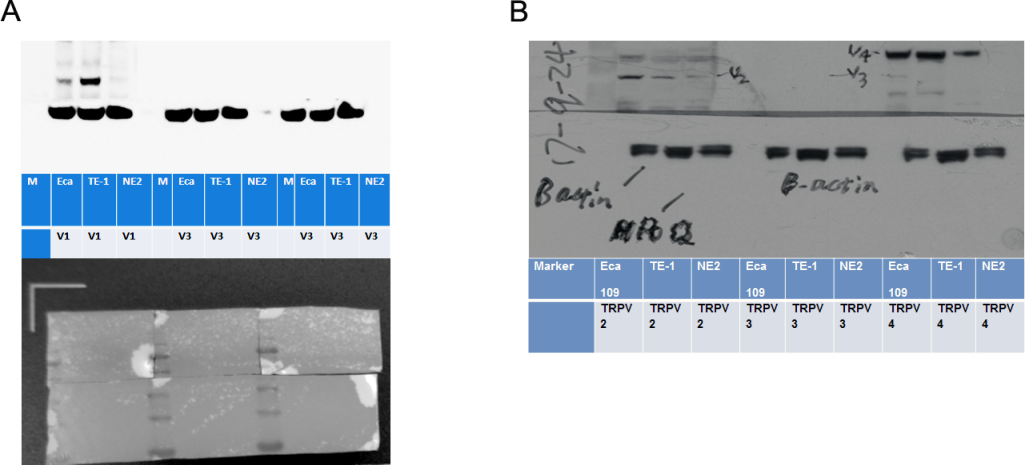
**

**Fig. S1. Raw western blot data.** Uncropped images of western blots in main figure 1B are shown. M: marker; Eca: Eca109; V1: TRPV1; V3: TRPV3

**
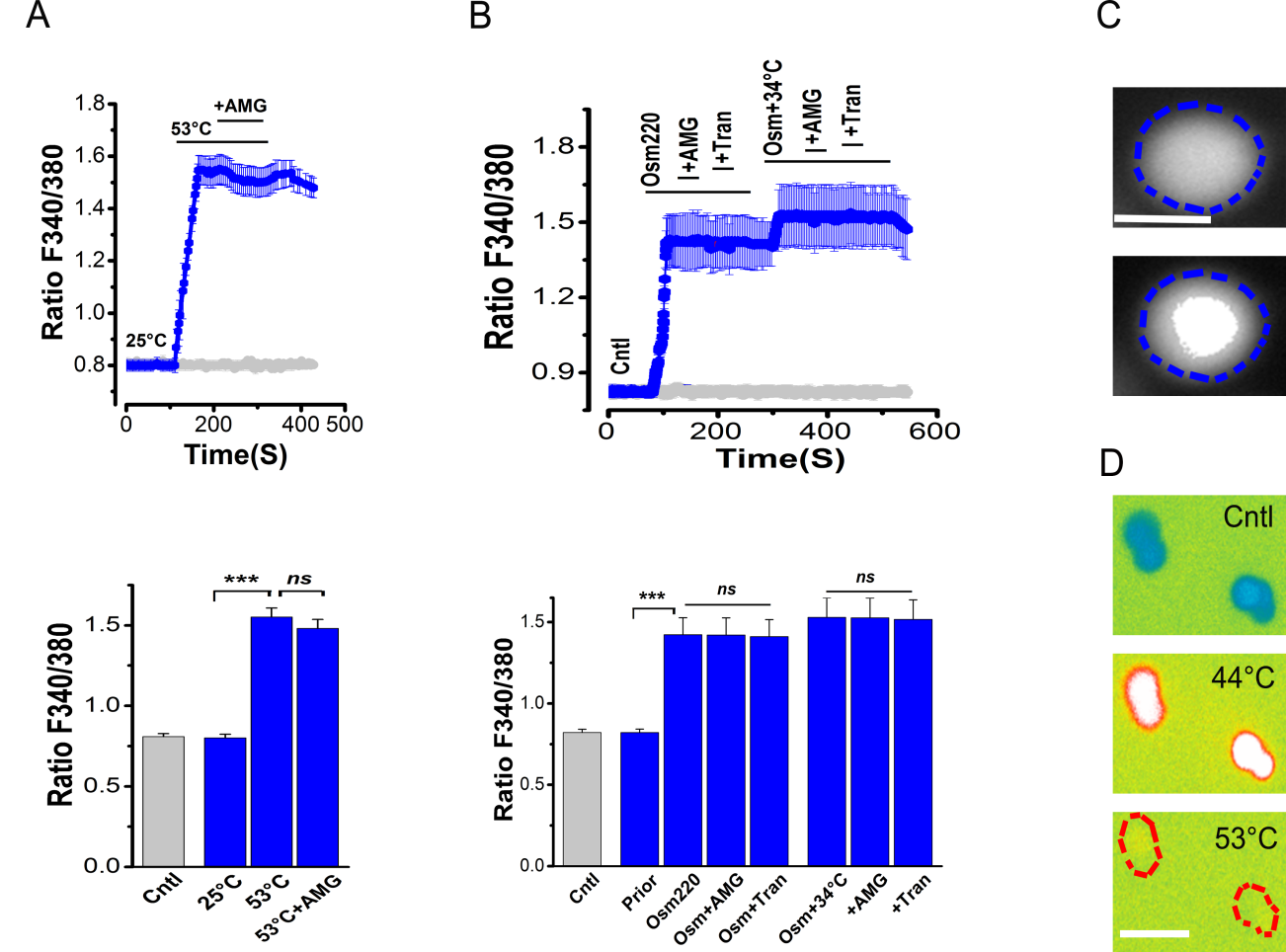
**

**Fig. S2. Activation of specific thermo-TRPVs in Eca109 cells. (A)** [Ca^2+^]_i_ was elevated considerably on the exposure to 53°C (the activation temperature threshold for TRPV2) and this effect was slightly influenced (*p* > 0.05) by the co-administration of AMG9810, a TRPV1 inhibitor. (n = 35-40). **(B)** [Ca^2+^]_i_ was enhanced substantially on the exposure to the hypotonic HBSS **(**220 m Osm) ; heat stimulation (34**°**C) potentiated the hypotonic effect and these effects were neither significantly affected by AMG9810 nor Tranilast (n = 30-40). **(C)** A cell was selected for recording by MetaFluor software during Ca^2+^ imaging measurement, pictures were captured at excitation wavelength 340nm. Upper panel: control; lower panel: exposure to 53**°**C HBSS, enhanced intensity of fluorescence_340nm_ was shown. **(D)** Representative cell images captured at ratio F340/380 during Ca^2+^ imaging assay, top: control; middle: exposure to 44**°**C; bottom: exposure to 53**°**C. Cntl: control; AMG: AMG9810; Tran: Tranilast; Prior: prior to treatment; Osm220: osmotic pressure 220 mm Hg.

*ns*: not significant; **** p < 0.001,* one-way ANOVA. Scale bar (in C and D): 10 μm.


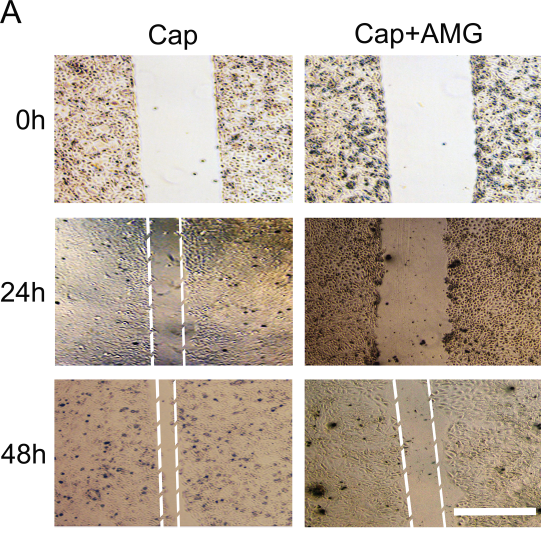


**Fig. S3.** **Effects of over-activation of TRPV1 by capsaicin on the migration of Eca109 cells.** Cell migration was assessed via a wound healing assay. **(A)** Representative images of Eca109 cell migration after exposure to capsaicin (15 μM) or capsaicin + AMG9810 (10 nM). The white dashed lines assisted to define the edging of the wounds. Scale bar: 1.0 mm.

**
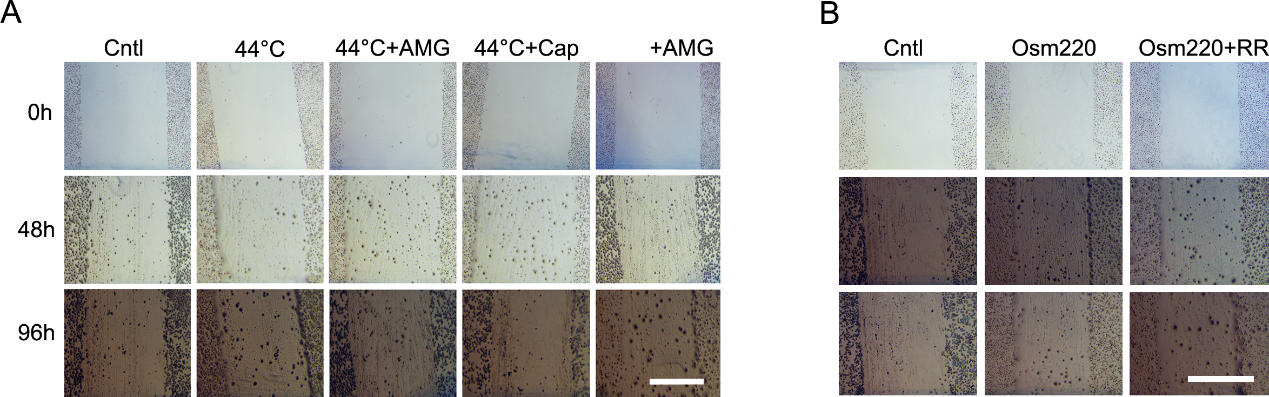
**

**Fig. S4. Impacts of over-activation of TRPV-1 and 4 on the migration of NE2 cells.** Cell migration was measured via a wound healing assay. **(A)** Exemplar images of NE2 cell migration after recurrently brief exposure to heat stimuli (44**°**C water bath) and application of capsaicin (15 μM). AMG9810 was used as a TRPV1 antagonist. **(B)** Representative pictures of NE2 cell migration after recurrently brief exposure to hypotonic media (220 m Osm). Ruthenium red (RR) was used as a TRPVs inhibitor. Cntl: control; Cap: capsaicin; AMG: AMG9810; RR: ruthenium red; Osm220: osmotic pressure 220 mm Hg. Scale bar: 500 μm.
